# Chromosome-Level Genome Announcement of the Monokaryotic *Pleurotus ostreatus* Strain PC80

**DOI:** 10.3390/jof11080563

**Published:** 2025-07-29

**Authors:** Jie Wu, Wenhua Sun, Jingkang Zheng, Jinling Liu, Xuedi Liang, Qin Liu, Weili Kong

**Affiliations:** Institute of Edible Fungi, Henan Academy of Agricultural Sciences, Key Laboratory of Evaluation and Utilization of Germplasm Resources of Edible Fungi in Huang-Huai-Hai Region, Ministry of Agriculture and Rural Affairs, Zhengzhou 450002, China

**Keywords:** *Pleurotus ostreatus*, whole genome, mating-type loci, genome annotation, CAZyme profiles

## Abstract

*Pleurotus ostreatus* is a widely cultivated edible fungus in China, renowned for its rich nutritional composition and diverse medicinal compounds. However, the quality of the currently published *P. ostreatus* genomes remained suboptimal, which limited in-depth research on its evolution, growth, and development. In this study, we conducted a chromosome-level genome assembly of the monokaryotic basidiospore strain PC80. The assembled genome spanned 40.6 Mb and consisted of 15 scaffolds. Ten of these scaffolds contained complete telomere-to-telomere structures. The scaffold N50 value was 3.6 Mb. Genome annotation revealed 634 carbohydrate-active enzyme (CAZyme) family genes. Through collinearity analysis, we further confirmed that the PC80 genome exhibited higher completeness and greater accuracy compared to the currently published genomes of *P. ostreatus*. At the *matA* locus of PC80, three *hd1* genes and one *hd2* gene were identified. At the *matB* locus, seven pheromone receptor genes and two pheromone precursor genes were detected. Further phylogenetic analysis indicated that three of these pheromone receptor genes are likely to have mating-specific functions. This complete genome assembly could provide a foundation for future genomic and genetic studies, facilitate the identification of key genes related to growth and developmental regulation, and promote technological innovations in *P. ostreatus* breeding and efficient utilization.

## 1. Introduction

*Pleurotus ostreatus* (oyster mushroom) belongs to the phylum Basidiomycota, class Agaricomycetes, order Agaricales, and family Pleurotaceae [1]. It is one of the most widely cultivated edible fungi worldwide, valued for its rich nutritional content, ease of cultivation, and medicinal properties, such as antioxidant, antitumor, and immunomodulatory effects [2,3]. As a typical white-rot fungus, *P. ostreatus* plays a vital ecological role as a key decomposer of lignocellulose in forest ecosystems [4]. Its lignin-degrading ability is of significant interest for applications in bioremediation, environmental protection, and sustainable agriculture [5,6].

The first genome sequencing of *P. ostreatus* was performed using Sanger sequencing, enabling the assembly of the genomes of the haploid derivatives PC9 and PC15 from the diploid strain N001 originating in Navarra, Spain [7,8]. In recent years, the genome data of PC9 have been updated through next-generation sequencing technologies [9]. The accumulation and improvement of these genomic resources have provided critical sequence information for the development of efficient genetic transformation systems and various selectable markers, thereby supporting the application of genetic manipulation techniques such as gene knockout and genome editing, and facilitating advances in studies on functional gene analysis and metabolic regulation mechanisms [4]. Regarding lignin degradation mechanisms, researchers have identified multiple ligninolytic enzyme genes [6,10], and the essential roles of these genes in lignin degradation have been confirmed through gene knockout and CRISPR/Cas9 technologies [11]. However, the current genome data still provide insufficient annotation of the genes encoding degradation enzyme families, regulatory factors, and secretion system components, which hinders the systematic analysis of the integrity of the degradation system [4]. In studies on sexual development regulation, CRISPR/Cas9 technology has been applied to analyze the functions of genes associated with sexual development (*pcc1* and *clp1*), revealing both the conservation and diversity of sexual development mechanisms between *Coprinopsis cinerea* and *P. ostreatus* [12]. Similarly, the lack of in-depth studies on the gene structures of mating-type loci and the inability to precisely map regulatory elements have made it difficult to construct key genetic materials, such as monokaryotic strains in which the A/B locus pathways are not activated [13,14]. Moreover, current genome assemblies of *P. ostreatus* are still fragmented, and complex structural regions such as repetitive sequences, regulatory elements, telomeres, and centromeres have not yet been fully resolved [8,9]. Most studies have focused on the PC9 strain alone, and the lack of high-quality genome comparisons among different strains and cultivars limits the deeper exploration of genomic diversity and functional variation. Therefore, a new high-quality genome is needed to support further research.

HiFi sequencing and Hi-C are two key technologies in modern genome assembly. HiFi sequencing, with its high accuracy and long reads, improves the quality of the initial assembly, while Hi-C provides chromosome-level spatial information to accurately anchor and refine the assembly [15,16]. This combination has been successfully applied to generate high-quality genomes in various species, such as *Rhododendron vialii* [17], *Eretmochelys imbricata* [18], and *Agrocybe chaxingu* [19].

In this study, we conducted a de novo chromosome-level genome assembly of the monokaryotic strain PC80, which was derived from the dominant cultivar “HeiKang 650” in Henan Province, by integrating Illumina, PacBio HiFi, and Hi-C technologies. Comparative genomic synteny analysis revealed issues in the assembly of previously reported *P. ostreatus* strains. Additionally, we performed a detailed analysis of the abundance and functional characteristics of the carbohydrate-active enzyme (CAZyme) family in the PC80 genome, and explored their potential roles in carbohydrate metabolism and lignocellulose degradation. Finally, by identifying homeodomain transcription factors, pheromone receptors, and pheromone precursors, we located the positions of *matA* and *matB*, and distinguished between mating type-specific and non-mating type-specific pheromone receptors in *matB*. Through chromosome-level assembly and mating-type loci analysis, this study provides the most complete *P. ostreatus* genome assembly to date and could enhance our understanding of the high variability of mating-type loci.

## 2. Materials and Methods

### 2.1. Fungal Cultivation

The dikaryotic *P. ostreatus* strain “Heikang 650” is characterized by high yield, strong resistance to environmental stresses, and uniform, synchronized fruiting body formation, making it an ideal parent strain for genome sequencing to support breeding and functional genomic studies. This strain was artificially cultivated at the Henan Modern Agricultural Research and Development Base, located in Yuanyang County, Xinxiang City, Henan Province, China. The cultivation substrate was composed of 98% cottonseed hulls and 2% lime, with a material-to-water ratio of 1:1.3. When the fruiting bodies matured (typically 30–35 days after inoculation), they were harvested, and basidiospores were collected under sterile conditions. The basidiospores were then transferred into sterile water for gradient dilution [20], and the diluted suspension was spread onto PDA plates for incubation. The tips of mycelia emerging from germinating basidiospores were excised, prepared as slides, and examined under a stereomicroscope (BX53F, Olympus, Tokyo, Japan). Mycelia that exhibited no clamp connections were considered monokaryotic and were subsequently transferred to fresh PDA plates for further cultivation. A randomly selected monokaryon was named “PC80” for subsequent experiments. The parent strain “HeiKang 650” and all monokaryons were preserved in the germplasm resource bank of the Edible Fungi Research Institute, Henan Academy of Agricultural Sciences, China.

### 2.2. Sequencing, Assembly, and Correction

Genomic DNA from PC80 was extracted using the cetyltrimethylammonium bromide (CTAB) method and its quality was assessed using Nanodrop, Qubit, and agarose gel electrophoresis [21].

The Illumina PE150 library was prepared using the NEBNext^®^ Ultra™ DNA Library Prep Kit (New England Biolabs, Ipswich, MA, USA). High-quality genomic DNA was randomly fragmented using a Covaris ultrasonicator (Woburn, MA, USA), followed by end repair, A-tailing, adaptor ligation, and PCR amplification. The sequencing was performed on the Illumina platform, yielding approximately 20.78 million paired-end reads. After adapter removal and quality filtering using fastp v0.23.4 [22], reads containing more than 10% ambiguous bases (N) or with over 20% of bases having a quality score ≤5 were discarded, resulting in approximately 7.2 Gb of high-quality clean data. The resulting data had a Q30 proportion of 89.77%, GC content of 49.05%, and a sequencing error rate of approximately 0.03%. These quality metrics were deemed sufficient for subsequent K-mer analysis and genome assembly polishing.

The PacBio library was constructed following the standard SMRTbell protocol. High-quality genomic DNA with a main band above 30 kb was sheared into 15–18 kb fragments using ultrasonication, followed by DNA damage repair, end repair, A-tailing, and adapter ligation. After purification and size selection, sequencing was performed on the PacBio Sequel II platform. The raw sequencing data were first processed to remove adapter sequences, resulting in subreads, which were then filtered based on a minimum length threshold of 50 bp. Subsequently, the subreads were processed using the CCS (https://github.com/PacificBiosciences/ccs, accessed on 10 October 2024) with parameters set to min-passes = 3 and min-rq = 0.99, yielding approximately 8.73 Gb of high-quality PacBio HiFi reads with an N50 of 19,033 bp.

Genome assembly was initially performed using Hifiasm with the PacBio HiFi long-read data [23]. The draft assembly was subsequently polished in two rounds using high-quality clean reads generated from the Illumina platform with NextPolish (https://github.com/Nextomics, accessed on 17 October 2024).

Illumina and PacBio HiFi sequencing were performed by Beijing Novogene Technology Co., Ltd. (Beijing, China). The de novo genome assembly was completed by the Edible Fungi Research Institute of the Henan Academy of Agricultural Sciences.

### 2.3. Chromosome Assembly by Hi-C

To achieve a chromosome-level genome assembly, fungal strain samples were used to construct a high-throughput chromosome conformation capture (Hi-C) library. The Hi-C procedure began with cell crosslinking using formaldehyde, followed by permeabilization to maintain nuclear integrity. The DNA was then digested with the *Mbo*I restriction enzyme, and the 5′-overhangs were filled in by incorporating a biotinylated nucleotide. Subsequently, the resulting blunt-end fragments were ligated, and their biotinylated ligation junctions were captured using streptavidin beads. In the final step, the captured fragments were analyzed by paired-end sequencing on the Illumina HiSeq X Ten platform (San Diego, CA, USA) using the PE-150 module, which is essential for mapping chromosomal interactions [24].

Quality control of the Hi-C data was performed with Hi-C Pro. Clean Hi-C reads were mapped to a reference genome assembled from PacBio and Illumina data using the Juicer tool. This mapping process produced the inter- and intrachromosomal contact maps, with the exclusion of low-quality reads. These Hi-C interactions provided critical information for determining the contig proximity. The 3D-DNA pipeline was used for further genome assembly [25], scaffolding the genome into 13 pseudochromosomes.

### 2.4. RNA Extraction and Sequencing

RNA was extracted using the TRIzol^®^ Reagent (Invitrogen, Carlsbad, CA, USA) following the manufacturer’s instructions. The samples were first freeze-dried and then thoroughly ground into a fine powder in liquid nitrogen. All extraction steps were carried out on ice or at 4 °C to prevent RNA degradation. After phase separation, the aqueous phase was recovered and further purified using the Plant RNA Purification Reagent (Invitrogen, USA). Residual genomic DNA was removed by treatment with RNase-free DNase I. The integrity and concentration of the RNA were assessed using an Agilent 2100 Bioanalyzer and a NanoDrop spectrophotometer, respectively. RNA library preparation was performed using the TruSeq Stranded mRNA Library Prep Kit (Illumina, San Diego, CA, USA). PE150 paired-end sequencing was conducted on the Illumina NovaSeq 6000 platform. A total of 53.01 million reads were generated from *P. ostreatus*, and 7.93 Gb of clean data was obtained after quality control by Trimmomatic (v0.39) [26]. All these procedures were performed by Beijing Novogene Technology Co., Ltd.

### 2.5. Genome Size Estimation

The genome size of *P. ostreatus* was estimated using GenomeScope v2.0 [27], which analyzed k-mer patterns (k = 21) from genomic Illumina short reads with the assistance of Jellyfish v2.3.1 [28].

### 2.6. Genome Completeness Assessment

This study used BUSCO v5.4.7 [29] to assess genome assembly completeness. For gene annotation, the basidiomycota_odb9 lineage dataset (n = 1706) was employed. Genome integrity was evaluated by aligning clean sequence reads. Specifically, Illumina short reads and PacBio HiFi long reads were mapped to the unmasked genome using BWA-MEME2 v2.2.1 [30] and minimap2 v2.26 [31], respectively. The HISAT2 v2.2.1 [32] was used to align RNA-Seq reads for the repeat-masked genome.

### 2.7. Genome Annotation

Using RepeatModeler v2.0.2 (https://github.com/Dfam-consortium/RepeatModeler/releases/tag/2.0.2a, accessed on 25 October 2024), a de novo prediction of repetitive sequences, including transposable elements (TEs), within the genome was performed. These predicted sequences were integrated with the RepBase database [33] and then combined with RepeatMasker v4.1.5 [34] to align, identify, and classify them.

Based on homology-based methods, a combination of de novo prediction and transcriptome strategies was employed to predict protein-coding genes. The transcriptional data, represented by Illumina reads, were mapped to the genomic sequences using HISAT2 [32]. Subsequently, the gene structures were described using BRAKER2 [35]. The predicted protein sequences were functionally annotated through comparison with several public databases, including Nr, KOG, SwissProt, KEGG, and Pfam. BUSCO was used to assess the accuracy of the gene predictions, using the basidiomycota_odb9 dataset as a reference.

### 2.8. Genomic Collinearity Analysis

We performed a comprehensive genome-wide alignment of PC80, PC9_AS, and PC15 using MUMmer v4.0.0 [36]. Based on this alignment, homologous gene pairs and their genomic coordinates were extracted and used to visualize genomic collinearity with the MCScanX toolkit implemented in Python v3.9.0 [37].

### 2.9. Sequence Analysis for the Mating-Type Locus

The mating-type genes in PC80 were identified based on homology with previously published counterparts in *C. cinereus* [38]. For the identified mating-type loci, conserved domain prediction was performed using CD-search [39] to confirm the presence of homeobox or STE3 domains. Transmembrane domain prediction of pheromone receptors was carried out using DeepTMHMM (https://dtu.biolib.com/DeepTMHMM, accessed on 2 November 2024) [40]. After identifying the pheromone receptor genes, short sequences ranging from 20 to 100 amino acids in the upstream and downstream regions were manually searched for potential pheromone precursor genes that contain conserved C-terminal CaaX and ER motifs [41].

All multiple sequence alignments were performed using the MUSCLE algorithm implemented in MEGA 12 [42], and alignment results were visualized with Jalview 2.11.4.1.

The receptor protein sequences used for phylogenetic tree construction were obtained from *Cryptococcus neoformans*, *C. cinerea*, *Schizophyllum commune*, *Pleurotus djamor*, *Lentinula edodes*, and *Flammulina velutipes* [41,43,44,45]. The corresponding sequences were retrieved from NCBI GenBank. The GenBank accession numbers can be found in Table S1. Phylogenetic tree construction was performed using the neighbor-joining (NJ) method in MEGA 12, with evolutionary distances calculated using the Poisson model. Bootstrap support was estimated based on 1000 replicates. Branches with bootstrap values above 70% were considered strongly supported, while those below 50% were not shown in the phylogenetic tree.

## 3. Results and Discussion

### 3.1. Genome Assembly of the P. ostreatus PC80 Strain

**Figure 1:**
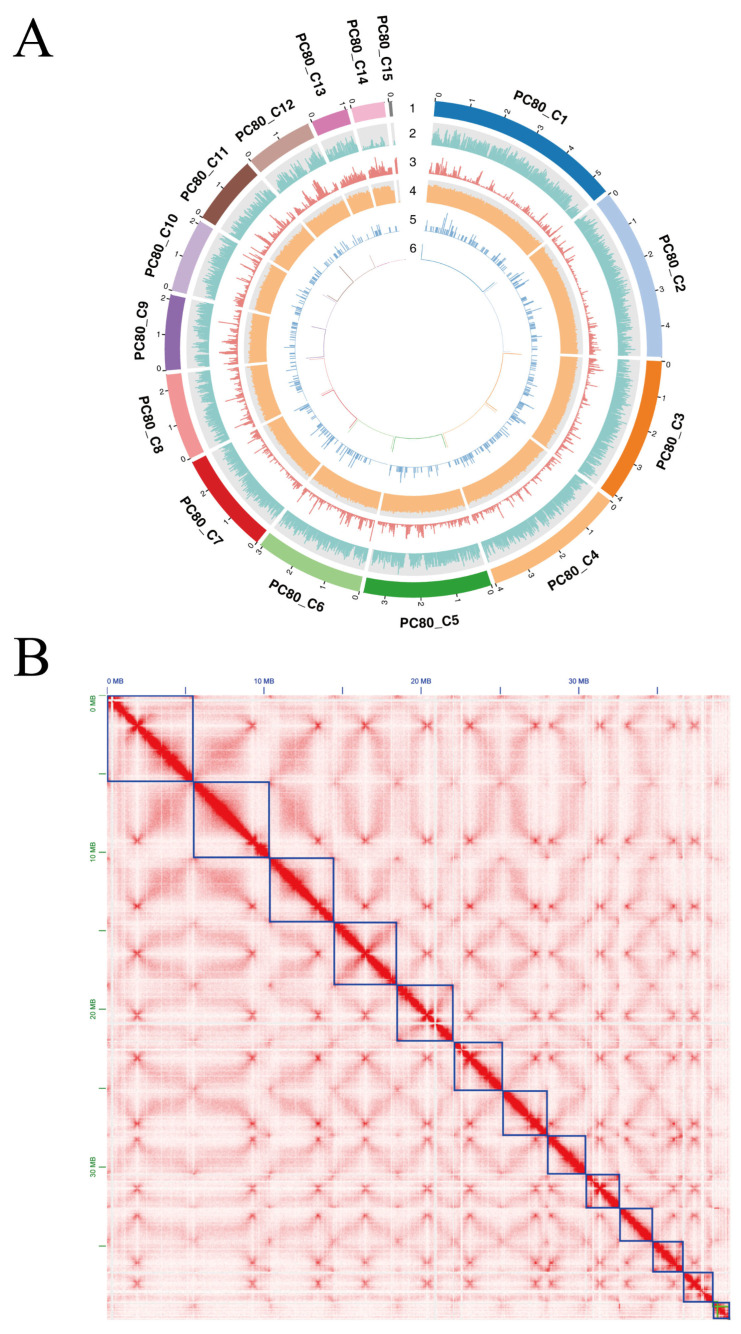
(**A**) Genome features of *Pleurotus ostreatus* strain PC80. The tracks (outer to inner) represent the distribution of genomic features assembly: (1) Sizes (in Mb) of PC80 scaffolds, with numbers prefixed by the letter “C” indicating the order of scaffold size; (2) Gene density; (3) Repetitive sequence density; (4) GC content; (5) Number of CAZymes; (6) Distribution of telomere repeats. Gene density, repeat sequence density, GC content, and CAZyme number distribution were all calculated using a 20 kb sliding window; (**B**) Hi-C heat map of the interaction between the genome chromosome of the PC80 genome.

Initially, approximately 7.2 Gb of clean Illumina short reads were used to estimate the genome size and polish the draft genome assembly. Based on a 21-mer analysis (Figure S1), the heterozygosity level of the PC80 strain was found to be only 0.07%. The estimated genome size was 40.7 Mb with 20.9% repetitive sequences. To enhance the quality of the *P. ostreatus* genome assembly, PacBio HiFi long reads were integrated with Illumina short reads, followed by Hi-C reads, which improved the assembly to a chromosome-level resolution. The final assembly of the *P. ostreatus* PC80 strain genome reached approximately 40.6 Mb, which closely matched the estimated genome size (Table 1). This assembly size surpassed those of previously published *P. ostreatus* genomes, such as PC9_AS (35.0 Mb), PC9_JGI (35.6 Mb), and PC15 (34.3 Mb) [8,9]. In comparison with other species within the genus *Pleurotus*, the assembly size was smaller than those of *Pleurotus tuoliensis* (48.2 Mb) and *P. eryngii* (49.9 Mb) [46,47], comparable to *Pleurotus giganteus* (40.0 Mb) [48], and larger than *Pleurotus tuber-regium* (35.8 Mb), *Pleurotus citrinopileatus* (36.8 Mb), and *Pleurotus platypus* (39.0 Mb) [49,50]. The genome sequence of PC80 was distributed across 15 scaffolds (Figure 1A), with the largest and smallest scaffold sizes being 5.52 Mb and 9.17 kb, respectively (Table S2). The smallest scaffold (PC80_sc15) corresponded to the mitochondrial genome. Moreover, the N50 of the PC80 genome was 3.60 Mb, the highest among the available *P. ostreatus* genomes, including PC9_AS (N50 = 3.50 Mb), PC9_JGI (N50 = 2.09 Mb), and PC15 (N50 = 3.27 Mb) (Table 1). To evaluate the quality and integrity of the assembly, we compared the sequencing data with the assembly results and found that the mapping rate was 99.87% and the BUSCO completeness was 98.8% (dataset: basidiomycota_odb10) (Figure S2), which were higher than those of other published *P. ostreatus* genomes (Table 1).

Hi-C, an advanced chromosome conformation capture method for detecting genome-wide chromatin interactions, has been widely applied to improve de novo assembled contigs into chromosome-level genome assemblies [51]. In this study, we performed Hi-C sequencing on the *P. ostreatus* PC80 strain, yielding 5.9 Gb of Hi-C reads after quality control. The contigs in the draft genome assembled from Illumina short reads and PacBio HiFi long reads were anchored and oriented using the Hi-C scaffolding method to generate a chromosome-scale assembly. The Hi-C contact map figures, particularly the centromeric interaction regions, were employed to determine the number of chromosomes [52]. Finally, the Hi-C matrix diagram showed that 13 clusters were successfully formed, each containing both intra-chromosomal and inter-chromosomal interactions. Based on this matrix, the genome assembly was oriented and anchored to 13 pseudo-chromosomes (Figure 1B), with chromosome lengths ranging from 1.04 Mb to 5.52 Mb and an anchoring rate of 97.46%.

The highly conserved sequence (TTAGGG)n is commonly used to identify telomere locations, representing the ends of chromosomes in fungi [53]. Except for the smallest mitochondrial genome scaffold (scaffold 15), among the remaining 14 scaffolds, 10 contained telomeric repeats at both ends, indicating that these scaffolds corresponded to the complete chromosomes, specifically chr01, chr02, chr03, chr04, chr05, chr06, chr07, chr08, chr09, and chr11. Scaffold 10 and scaffold 12 had telomeric repeats at one end, while scaffolds 13 and 14 did not exhibit telomeric repeats at either end (Figure S3). The length of the telomeric sequence ranged from 90 to 264 nucleotides (Table S3). Due to limited experimental conditions, Larraya et al. [7] only detected 11 chromosomes in PC9 and PC15 using pulsed-field gel electrophoresis in 1999. This was the earliest report on chromosome research of *P. ostreatus*. However, the advanced Hi-C sequencing data from our study identified 13 pseudo-chromosomes in PC80, representing the most complete genome in *P. ostreatus* to date. The assembly is closest to the 11 chromosomes reported by Larraya et al., with high quality and integrity.

### 3.2. Repeat Annotation and Genome Prediction

In the genome assembly of strain PC80, we identified 5.83 Mb of repetitive sequences, which accounted for 14.35% of the genome (Table 2). Among the annotated repetitive sequences, transposable elements represented the majority. Based on their structural features, transposable elements were divided into Class I (retrotransposons) and Class II (DNA transposons). Within Class I, the LTR/Gypsy family was the most abundant, comprising 4.79% (1.95 Mb) of the genome. In Class II, the DNA/CMC-EnSpm family was predominant, accounting for 0.33% (0.13 Mb) of the genome. Transposable elements, as mobile genetic elements, served as a significant source of genetic variation and evolutionary innovation in many organisms [54]. For example, collinearity analysis between the PC9 and PC15 genomes revealed that some regions lacking homologous sequences were replaced by LTR/Gypsy and DNA/CMC-EnSpm elements [55]. The density and distribution of repetitive sequences in PC80 were highly variable (Figure 1A). Regions rich in repetitive sequences were mostly situated in gene-sparse regions. In fungi, centromeres are typically characterized by the accumulation of transposable elements [38]. The centromere positions of the ten scaffolds with two telomeres were identified using Hi-C heatmaps. The regions near the inferred centromeres showed a high density of repetitive sequences (Figure 1).

Multiple databases were used to ensure the accuracy of genome annotation, resulting in 14,374 genes annotated in the PC80 genome (Table 3). The gene count per scaffold was detailed in Table S4. The majority of annotated genes were matched to the Nr database (12,917 genes), followed by Pfam (7980 genes), SwissProt (6459 genes), KOG (4217 genes), and KEGG (4261 genes). The number of annotated genes in PC80 were significantly higher than those in PC9_AS (11,875 genes), PC9_JGI (12,206 genes), and PC15 (12,330 genes) [9]. Additionally, BUSCO evaluation yielded a score of 99.0% (Figure S4), underscoring the high accuracy and completeness of the *PC80* genome annotation.

The top 20 species that were matched in the Nr database exhibited a distinct distribution pattern (Figure S5). Only the top six species exhibited relatively high numbers of matched genes: *Cyclocybe pediades* (1495), *Cyclocybe aegerita* (1478), *Amanita inopinata* (1026), *Agaricus bisporus* (551), *Amanita brunnescens* (518), and *Amanita muscaria* (479). Subsequently, the number of matched genes sharply declined to as few as 1 to 3 genes per species. The top six species and *P. ostreatus* all belonged to the order Agaricales but did not fall under the family Pleurotaceae or the genus *Pleurotus*. On the one hand, this finding reflected the extensive sharing of highly conserved gene sequences among Agaricales species; on the other hand, it underscored the insufficient annotation completeness of *Pleurotus* species in the NR database.

**Figure 2:**
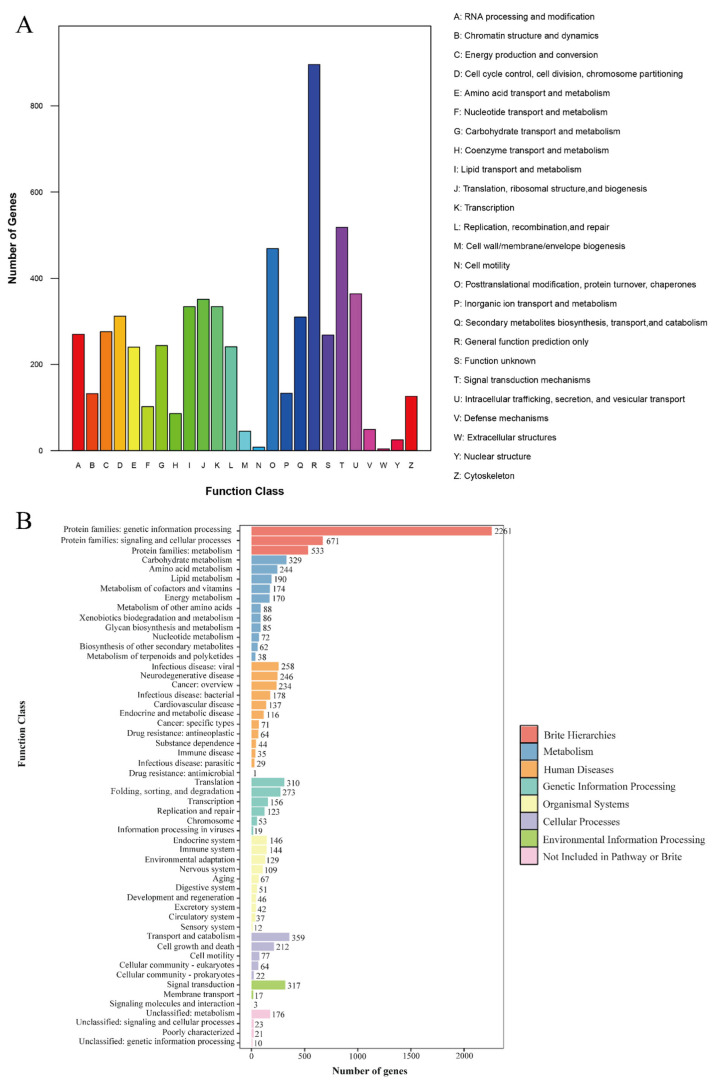
PC80 genome functional annotation analysis: (**A**) Distribution of KOG functional categories and gene count statistics; (**B**) KEGG biological domain classification and gene distribution.

In the KOG mapping of 4217 genes (Figure 2A), the largest group was assigned to the “General function prediction only (R)” category (896 genes), suggesting that although these gene products might have performed certain functions, their specific biological roles remained to be elucidated. This was followed by the “Signal transduction mechanisms (T)” category (518 genes) and the “Posttranslational modification, protein turnover, chaperones (O)” category (469 genes). These three categories were also among the major annotated groups in PC9_JGI and PC15, with the R category comprising 761 and 1076 genes, the T category 494 and 667 genes, and the O category 468 and 662 genes, respectively (Table S5). In parallel, KEGG analysis classified 4261 genes into eight hierarchical levels across 54 pathways (Figure 2B). This number was slightly lower than those observed in PC9_JGI (4473 genes) and PC15 (4697 genes) (Table S5), which may be attributed to strain-specific differences as well as variations in genome assembly. Specifically, in the “Brite hierarchies” subgroup of PC80, 2261, 671, and 533 genes were assigned to genetic information processing, signaling and cellular processes, and metabolism, respectively. Notably, the “Metabolism” category further encompassed 1538 genes that were involved in key physiological functions such as carbohydrate, energy, and amino acid metabolism. The integration of functional predictions from KOG with pathway information provided by KEGG indicated that PC80 harbored a complex regulatory network underpinning essential processes, including cellular replication, gene expression, signal transduction, and energy and material conversion. This complexity also underscored the adaptive capacity of saprotrophic fungi, which possessed highly sensitive signal transduction systems that enabled rapid responses to environmental changes in decaying wood, leaf litter, and soil [56].

### 3.3. Synteny Analysis Between P. ostreatus PC80 and PC9_AS

**Figure 3:**
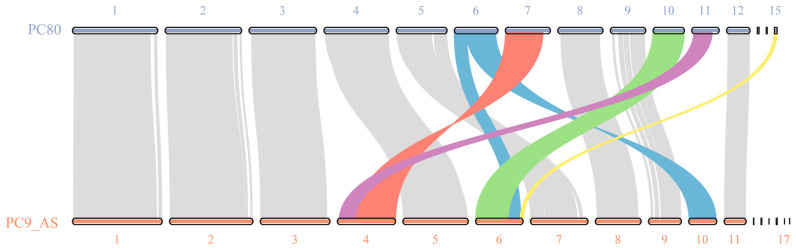
Genome collinearity between PC80 and PC9_AS. Different colored lines represent the correspondences between PC80 and PC9_AS involving different numbers of scaffolds: the gray lines denote one-to-one collinearity between scaffolds, while the blue, red, green, purple, and yellow lines indicate collinearity among multiple scaffolds.

To further verify the assembly accuracy of the PC80 genome, we performed a synteny analysis between PC80 and the latest publicly available *P. ostreatus* genome version, PC9_AS (Figure 3). The results revealed a high degree of collinearity between PC80 and PC9_AS. For example, scaffolds 1, 2, 3, 4, 5, 8, 9, and 12 of PC80 corresponded almost perfectly to scaffolds 1, 2, 3, 5, 7, 8, 9, and 11 of PC9_AS. PC9_AS scaffold 4 showed collinearity with PC80 scaffolds 7 and 11, and all three were complete chromosomes with telomeres at both ends, indicating that PC9_AS may have erroneously merged the originally separate scaffolds 7 and 11 of PC80 into a single scaffold 4. Furthermore, PC9_AS scaffold 6 lacked telomeric structures at both ends, yet it was collinear with PC80 scaffolds 6, 10, and 15. In PC80, scaffold 6 possessed complete telomeres, and scaffold 15 had been confirmed as the mitochondrial genome. Notably, PC80 scaffold 6 was collinear not only with PC9_AS scaffold 6 but also with its scaffold 10, which further suggested that PC9_AS may have mistakenly merged sequences that should have been present as separate scaffolds 10, 15, and parts of scaffold 6 into a single scaffold 6 during assembly. We also included a synteny analysis between PC80 and PC15 (Figure S6). Although PC15 was assembled using the earlier Sanger shotgun method and exhibits limited completeness and continuity, it still demonstrated substantial collinearity with PC80. For instance, PC80 scaffold 11, which possesses telomeric sequences at both ends, corresponded only to a portion of PC15 scaffold 11, indicating that the latter may represent an incomplete chromosomal assembly.

The Hi-C heatmap confirmed the accuracy of the independent chromosomal structures in PC80 that exhibited complete telomeres. Based on a comprehensive comparison, we inferred that one reason for the larger genome size of PC80 compared to PC9_AS was that early genome assembly accuracy was limited by sequencing technology, which had an insufficient capacity to identify repeated sequences and led to the fragmentation of the complete sequence. This view was best reflected by the number of identified telomeric structures. Another reason was that basidiomycete species often exhibited variations in the number of repetitive elements. According to the assembly results, PC80 evidently contained a greater number of repetitive sequences.

### 3.4. The Carbohydrate-Active Enzyme Family

In fungal genomes, CAZyme genes encode enzymes involved in carbohydrate metabolism, degradation, and conversion processes [57,58]. This enables them to break down lignocellulose into simple sugars or other utilizable nutrients and provide energy for hyphal growth. CAZymes are mainly divided into six major modules: glycoside hydrolases (GHs), glycosyltransferases (GTs), auxiliary activity enzymes (AAs), polysaccharide lyases (PLs), carbohydrate esterases (CEs), and carbohydrate-binding modules (CBMs) [59]. The genome sequence of PC80 was analyzed using the CAZymes database, identifying a total of 634 genes encoding CAZymes, including 246 GHs, 52 GTs, 53 PLs, 31 CEs, 169 AAs, and 83 CBMs. The number of CAZyme genes in PC80 was higher than that reported for other *Pleurotus* species (Table 4), such as *P. giganteus* [48], *P. tuoliensis*, and *P. eryngii* [46].

In PC80, GHs (246 genes) and AAs (169 genes) dominated. Among the GHs in PC80, many families exhibited high copy numbers, such as GH3 (13 copies), GH5 (31 copies), GH7 (15 copies), GH13 (13 copies), GH16 (27 copies), and GH18 (14 copies). GH3, GH5, and GH7 might cooperatively degrade cellulose through a cascade mechanism. GH5 (endoglucanase) cleaves long cellulose chains [60], GH7 (cellobiohydrolase) releases cellobiose from cellulose chain ends [61] and GH3 (β-glucosidase) hydrolyzes cellobiose into glucose [47], thereby completing cellulose degradation. Additionally, GH13 (α-amylase) hydrolyzes starch and glycogen to produce maltose and glucose [62]. GH16 (β-1,3-glucanase) and GH18 (chitinase) participate in cell wall metabolism, regulating remodeling during growth and promoting fruiting body development [63]. Within the AA family, AA1 (12 copies), AA3 (61 copies), AA5 (24 copies), AA7 (23 copies), and AA9 (30 copies) showed high copy numbers. The AA1 family belongs to multicopper oxidases and serves as a hallmark enzyme for lignin degradation in white-rot fungi [47]. AA9 is a copper-dependent polysaccharide monooxygenase believed to act directly on cellulose and enhance the hydrolytic efficiency of GHs [64]. AA3 (GMC oxidoreductases) and AA5 (glyoxal oxidase, galactose oxidase) generate H_2_O_2_ by oxidizing glucose and alcohols, thus providing a cofactor for lignin degradation [65,66]. AA7 enzymes (flavin-containing monooxygenases) may be involved in the biotransformation or detoxification of lignocellulosic compounds [64]. Excluding GHs and AAs, many families in other modules showed relatively high copy numbers: PL1 (21), PL14 (13), CE4 (12), CBM1 (42), and CBM13 (19). PL1 (pectate lyase) and PL14 (polysaccharide lyase) degrade pectin and complex polysaccharides [67]. They target pectin in plant cell walls and establish a weak parasitic relationship with Apiaceae plants [68]. CE4 (chitin deacetylase, xylan esterase) facilitates xylan and chitin degradation through deacetylation [69]. Meanwhile, CBM1 (carbohydrate-binding module) and CBM13 (polysaccharide-binding module) bind to crystalline cellulose, xylan, and pectin, enhancing enzyme–substrate interactions [70]. Additionally, families such as GH27 (6), GH31 (7), GH35 (4), and GH10 (4) had relatively lower copy numbers and participated in hemicellulose degradation [71]. In PC80, the overall trend in CAZyme family copy numbers was consistent with the trend in PC9 and PC15 (Table S6). Families with high copy numbers in PC9 and PC15 also showed high copy numbers in PC80. Minor numerical differences were likely due to variations in genome assembly techniques and annotation methods. However, the GT2 family was an exception. It was detected with 10 and 12 copies in PC9 and PC15, respectively, but was not found in PC80.

### 3.5. Identification of the Mating Locus

*P. ostreatus* is a tetrapolar basidiomycete possessing two distinct mating loci, *matA* and *matB* [72]. In terms of function, the *matA* locus encodes two types of homeodomain transcription factors: HD1 and HD2, which control synchronous nuclear division and clamp cell formation [14]. The *matB* locus encodes pheromone precursors and pheromone receptor genes [73], which control nuclear migration and the fusion of clamp cells with subterminal cells [74,75]. In terms of sequence and structure, both *matA* and *matB* loci exhibit a high degree of diversity. For instance, in *C. cinerea*, the *matA* locus carries three pairs of functional *HD1-HD2* genes, with each pair encoding two types of homeodomain transcription factors (HD1 and HD2) [76]. These three gene pairs share a common evolutionary origin and exhibit a similar structural organization, but they have diverged significantly in sequence to avoid recombination and to facilitate the evolution of functionally distinct paralogous pairs [77,78,79,80]. Thus, the canonical matA allele (the “A archetype”) is believed to comprise three paralogous *HD1-HD2* gene pairs [77,81]. However, in other basidiomycetes, the *matA* locus structure rarely contains three complete gene pairs. The pheromone receptors encoded by *matB* are conserved in their structure [82]. The pheromone receptors in basidiomycetes are homologous to the a-factor receptor, like Ste3p in ascomycetous yeasts [14]. Pheromone precursors are generally composed of more than 20 amino acid residues, but the gene sequences encoding these precursors are short and highly variable [41]. However, the amino acid sequences of pheromone precursors often contain highly conserved functional motifs (such as the CaaX motif, EA motif, and AF motif) that serve as recognizable features. Among these, the most easily identifiable is the CaaX motif located at the C-terminus, where “C” represents cysteine, “a” stands for an aliphatic amino acid residue, and “X” can be alanine, serine, methionine, glutamic acid, or cysteine [83]. Only pheromones that are specific to different mating types can successfully bind to the pheromone receptors that are specific to the other mating type, thereby activating the associated G protein-coupled signaling cascade [84].

#### 3.5.1. *matA* Locus

**Figure 4:**
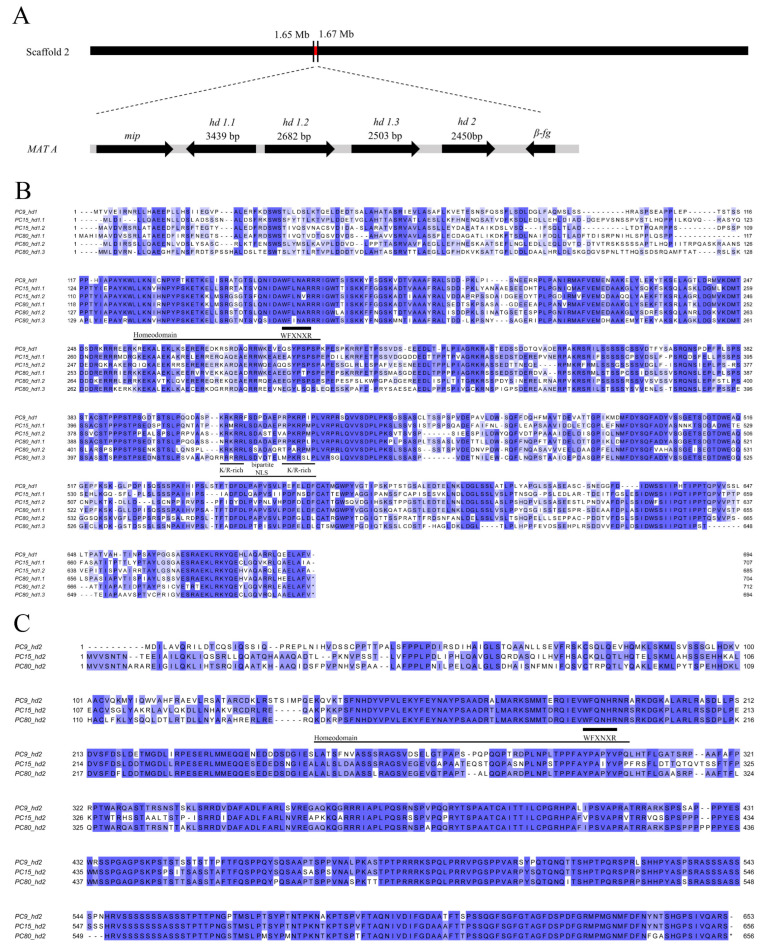
The *matA* locus and its gene products. (**A**) Gene structure of the chromosomal region containing the *matA* locus in the PC80 strain, showing the genomic position of *matA*, the size, and transcriptional orientation of the *hd* genes, as well as the adjacent *mip1* gene (encoding mitochondrial intermediate peptidase) and *β-fg* gene (encoding a conserved fungal protein of unknown function). (**B**,**C**) Protein sequence alignments of *hd* gene products from PC9, PC15, and PC80 strains. The alignments highlight the homeodomain regions, the conserved DNA-binding motif WFXNXR, and the positions of bipartite nuclear localization signals (NLSs).

The *matA* locus of PC80 was composed of three *hd1* genes (*hd1.1*, *hd1.2*, *hd1.3*) and one *hd2* gene (Figure 4A), containing multiple introns and exons (Table S7). The *matA* locus spanned a total length of 11,619 bp and was located on scaffold 2 of PC80. The three *hd1* genes measured 3439 bp, 2682 bp, and 2503 bp, respectively. *hd1.1* and *hd1.2* were arranged in a “head-to-head” orientation, while *hd1.2* and *hd1.3* were arranged in a “head-to-tail” configuration. Upstream of *hd1.1* was the *mip* gene, which encoded the mitochondrial intermediate peptidase. The *hd2* gene measured 2450 bp and was followed by the *β-fg* gene carrying a conserved sequence encoding a protein of unknown function. A similar pattern of *hd1* gene copies at the *matA* locus was also found in PC9 and PC15. These two homokaryotic strains were derived from the parental strain N001 through monokaryotization [72]. The *matA* locus in PC15 contained an additional *hd1* gene compared to PC9 (Figure S7). Notably, the *matA* locus in PC80 contained two more *hd1* genes than PC9.

Subsequently, we systematically compared the CDS and protein sequences of the *hd* genes from three monokaryotic strains: PC9, PC15, and PC80. Based on CDS sequence alignment (Figures S8 and S9), the *hd2* genes showed higher sequence identity than the *hd1* genes. The sequence identity of *hd2* genes across strains was above 74%. The highest sequence identity was 79.54% between *PC15_hd2* and *PC80_hd2*. In contrast, the CDS sequence identity of *hd1* genes was generally lower, mostly ranging from 62% to 73%. The highest sequence identity was 73.01% between *PC9_hd1* and *PC80_hd1.1*. Most of the other sequence comparisons showed approximately 65% identity (Tables S8 and S9). Based on protein sequence alignment, HD2 proteins were more conserved than HD1 proteins across the three strains. All HD2 protein sequences showed amino acid identities above 71%. The highest identity was 77.19% between *PC15_hd2* and *PC80_hd2*. In contrast, HD1 protein sequences exhibited lower identity levels, with most values below 60%. The highest sequence identity was 67.56% between *PC9_hd1* and *PC80_hd1.1*, while most other sequence identities ranged from 50% to 58% (Tables S10 and S11).

Previous studies in the model fungus *C. cinerea* showed that HD1 and HD2 homeodomain proteins at the *matA* locus contained three α-helical regions. The third helix carried a conserved WFXNXR motif [14]. HD1 proteins also contained nuclear localization signals, which ensured the nuclear import of HD1/HD2 heterodimers [73]. The N-terminal region of both proteins served as the dimerization domain and showed high sequence variation among strains [73]. This variation was also observed in the protein sequences of PC9, PC15, and PC80 (Figure 4B,C). In addition, all three strains retained highly conserved DNA-binding domains in their HD1 and HD2 proteins. The homeodomain region of HD2 proteins consisted of 54 amino acid residues, which was significantly longer than the 39-residue region in HD1 proteins. The WFXNXR motif was located between residues 152 and 176 in HD1 proteins and between residues 186 and 195 in HD2 proteins. The C-terminal region of HD1 proteins (residues 405–446) contained a bipartite nuclear localization signal.

In *C.cinereus*, transformation experiments demonstrated that only one pair of complementary HD1 and HD2 proteins forming a heterodimer was required to initiate the *matA* regulated sexual developmental process [77]. In PC80, all three *hd1* genes encoded proteins containing both a homeodomain and a bipartite NLS. All of them were transcriptionally active (Table S12). Therefore, it remained unclear which *hd1* gene was functionally expressed in PC80.

#### 3.5.2. *matB* Locus

**Figure 5:**
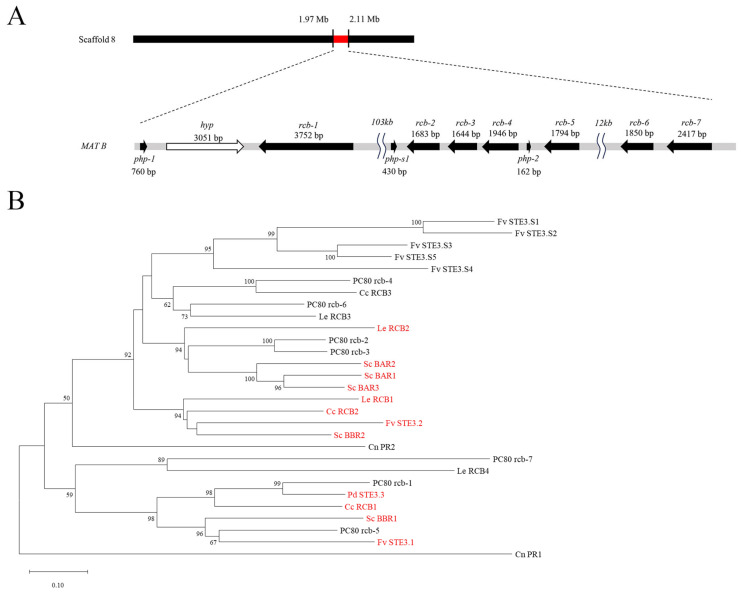
The *matB* locus and its gene products. (**A**) Gene structure of the *matB* locus in the PC80 strain, showing its chromosomal position, as well as the lengths and transcriptional orientations of pheromone receptor genes (*rcb-1* to *rcb-7*), pheromone precursor genes (*php-1* and *php-2*), a pheromone-like gene (*php-s1*), and a gene encoding a hypothetical protein (*hyp*). (**B**) Phylogenetic tree of pheromone receptors from PC80 and multiple other basidiomycete species. The tree was constructed using the neighbor-joining (NJ) method with 1000 bootstrap replicates. Nodes with bootstrap support ≥70% are considered statistically well supported; values ≥50% are also indicated. Fungal species are abbreviated in parentheses: *Pleurotus ostreatus* (PC), *Lentinula edodes* (Le), *Coprinopsis cinerea* (Cc), *Schizophyllum commune* (Sc), *Flammulina velutipes* (Fv), *Pleurotus djamor* (Pd), and *Cryptococcus neoformans* (Cn). Receptors known to have mating-type specificity are marked in red.

Seven distinct pheromone receptor genes were identified on scaffold 8 of the PC80 genome, distributed across a region of approximately 136 kb and sequentially named *rcb-1* to *rcb-7* (Figure 5A). Among them, *rcb-1* (3752 bp) was the longest gene in this region and was located most upstream; approximately 103 kb downstream, *rcb-2* (1683 bp), *rcb-3* (1644 bp), *rcb-4* (1946 bp), and *rcb-5* (1794 bp) were arranged in order. Further downstream by approximately 12 kb, *rcb-6* (1850 bp) and *rcb-7* (2417 bp) were identified sequentially. By manually searching the upstream and downstream regions of the identified receptor genes on scaffold 8, two putative pheromone precursor genes, named *php-1* and *php-2*, were further identified. They encoded proteins consisting of 52 and 53 amino acids, respectively. Both precursor genes contained a conserved MDS/IF motif at the N-terminus [85,86] and charged dipeptide motifs such as ER, EE, and DR in the middle region, which were considered as recognition sites for N-terminal processing of mature pheromones [87]. At the C-terminus, both contained a typical CaaX motif [88] (Figure S10). Genomically, *php-1* was located upstream of *rcb-1*, separated by a 3051 bp gene of unknown function. *php-2* was located between *rcb-4* and *rcb-5* (Figure 5A). In addition, we identified another candidate pheromone gene in the immediate upstream region of *rcb-2*, named *php-s1*, which encoded a protein of 74 amino acids. This gene shared 89.66% amino acid sequence identity with the pheromone gene *php3.2* from *Pleurotus eryngii var. eryngii* (GenBank Accession No.: AHL45288.1). *php-s1* also contained a conserved MDTF motif at the N-terminus and an ER motif in the middle region, but notably lacked the C-terminal CaaX motif (Figure S11).

In *C. cinerea*, the pheromone receptors encoded at the *matB* locus were classified as members of the fungal mating-type pheromone receptor Ste3 subfamily. They were full-length membrane proteins that contained seven transmembrane domains, including three intracellular loops and three extracellular loops, with the N-terminus located extracellularly and the C-terminus located intracellularly, where it interacted with G proteins [89,90,91]. We therefore performed conserved domain prediction analysis of the protein sequences of *rcb-1* to *rcb-7* using the NCBI Batch CD-search tool [39]. The results showed that all seven genes matched the STE3 superfamily (superfamily accession: cl12261) (Table S13). Among them, *rcb-1* to *rcb-6* exhibited strong homology with the STE3 superfamily, with E-values less than 1 × 10^−100^. Although *rcb-7* also matched the STE3 superfamily, its E-value was significantly higher (3.90 × 10^−67^), and it simultaneously matched a domain from the PHA03379 (EBNA-3A) superfamily, suggesting that it may function as a non-mating-specific pheromone receptor. We then predicted the transmembrane domains of *rcb-1* to *rcb-7* using DeepTMHMM [40]. The results showed that, except for *rcb-3* which contained only six transmembrane domains, the remaining six pheromone receptors all possessed a complete set of seven transmembrane domains (Table S14). The N-terminus of *rcb-3* was predicted to be intracellular and consisted of only one amino acid. To further investigate the evolutionary relationships among these pheromone receptor genes, we aligned their protein sequences (Figure S12). The results showed that *rcb-2* and *rcb-3* had the highest sequence identity at 73.30%. The sequence identity between other gene pairs was relatively low, mostly below 50%, such as 29.01% between *rcb-1* and *rcb-4*, and only 22.65% between *rcb-4* and *rcb-7* (Table S15). The phylogenetic tree constructed subsequently (Figure S13) showed that the seven pheromone receptor genes were divided into several distinct clades. Among them, *rcb-2* clustered with *rcb-3*, *rcb-4* clustered with *rcb-6*, *rcb-1* clustered with *rcb-5*, and *rcb-7* formed an independent branch. These results suggest that *rcb-3* may have originated from a duplication of *rcb-2*, but a sequence deletion at its N-terminus resulted in the loss of one transmembrane domain, indicating that it may also function as a non-mating-specific pheromone receptor.

Finally, we conducted a phylogenetic analysis of *rcb-1* to *rcb-7*, along with other known mating-specific and non-mating-specific pheromone receptor sequences from Basidiomycetes [41,43,44,45] (Figure 5B; mating-specific receptors were marked in red). The phylogenetic tree revealed that the PC80 receptor genes were distributed across two major clades. Among them, PC80 rcb-4 clustered with the non-mating-type receptor Cc RCB3, and PC80 rcb-6 clustered with the non-mating-type receptor Le RCB3; both were grouped within a large clade that also included non-mating-specific receptors Fv STE3.S1 to Fv STE3.S6. PC80 rcb-2 and PC80 rcb-3 formed a separate clade, grouping with the known mating-type-specific receptors Le RCB2 and Sc BAR1 to BAR3. PC80 rcb-7 clustered with the non-mating-type receptor Le RCB4. In addition, PC80 rcb1 clustered with the known mating-specific receptors Pd STE3.3 and Cc RCB1, and PC80 rcb-5 clustered with Sc BBR1 and Fv STE3.1, all of which have been confirmed as mating-type-specific receptors.

Pheromone receptor genes and pheromone precursor genes at the *matB* locus typically exhibit a characteristic clustering pattern [85]. Therefore, it can be inferred that *rcb-6* and *rcb-7*, which lacked neighboring pheromone precursor genes, were unlikely to be mating-type-specific receptors. Although *rcb-3* clustered with *rcb-2*, it was likely a duplication of *rcb-2* and lacked one transmembrane domain, indicating it also did not possess mating function. In summary, only *rcb-1*, *rcb-2*, and *rcb-5* simultaneously fulfilled two criteria: clustering with known mating-type-specific pheromone receptors and being located near pheromone precursor genes. Thus, they were the most likely candidates for mating-type-specific pheromone receptors.

## 4. Conclusions

In this study, we successfully assembled the chromosome-level genome of *P. ostreatus* strain PC80 by integrating Illumina, PacBio HiFi, and Hi-C sequencing technologies. The final assembly spanned 40.6 Mb and exhibits high completeness (BUSCO score of 98.8%) and continuity (N50 of 3.60 Mb), outperforming all previously published *P. ostreatus* genomes. The comprehensive annotation of repetitive sequences and synteny analysis with PC9_AS revealed that the larger genome size of PC80 could be attributed to the improved resolution of repetitive elements and more accurate identification of telomeric structures. A total of 14,374 protein-coding genes were annotated, including 634 CAZyme genes involved in lignocellulose degradation, highlighting the strain’s potential for biomass conversion. In addition, we conducted in-depth analysis of mating-type loci. At the *matA* locus, three *hd1* genes and one *hd2* gene were identified. The *matB* locus contained seven pheromone receptor genes and two pheromone precursor genes. Phylogenetic and structural analyses indicated that three of these receptor genes were likely to function as mating-type-specific receptors. Taken together, this work presented the most complete and functionally annotated genome of *P. ostreatus* to date and provided a valuable genomic resource for studying genome evolution, mating system complexity, and substrate utilization in saprotrophic fungi.

## Figures and Tables

**Table 1:** Genomic features of PC80 and its comparison with PC9_AS, PC9_JGI, and PC15.

General Features	PC80	PC9_AS	PC9_JGI	PC15
Total, nt	40,638,700	35,032,978	35,630,309	34,342,730
Number of scaffolds	15	17	572	12
N50 scaffold size, nt	3,609,302	3,500,734	2,086,289	3,270,165
Scaffold max. nt	5,521,148	4,859,873	4,430,591	4,830,258
Scaffold min. nt	91,722	9086	2001	280,724
GC content, %	50.94	50.79	50.94	50.95
BUSCO completeness, %	98.8	97.2	97.2	97.6

nt, nucleotides.

**Table 2:** Classification and quantity of repetitive sequences in the PC80 genome.

Classification	Number	Length	Percentage
CLASS I			
LINE/R1	21	140,592	0.34%
LINE/R2	4	19,595	0.04%
LINE/Tad1	14	8801	0.02%
LTR/Gypsy	630	1,947,966	4.79%
LTR/Unknown	690	261,661	0.64%
LTR/Copia	121	201,957	0.49%
LTR/Ngaro	12	16,302	0.04%
LTR/Pao	16	1969	0.00%
Total Class I repeat	1509	2,598,467	6.39%
CLASS II			
DNA tran/CMC-EnSpm	28	136,891	0.33%
DNA tran/Kolobok-H	11	92,152	0.22%
DNA tran/Zisupton	37	109,481	0.26%
DNA tran/Maverick	13	55,281	0.13%
DNA tran/P-Fungi	10	46,557	0.11%
DNA tran/MULE-MuDR	36	43,424	0.10%
DNA tran/TcMar-Sagan	35	25,931	0.06%
DNA tran/PIF-Harbinger	33	20,722	0.05%
DNA tran/TcMar-Tc1	17	10,182	0.02%
DNA tran/TcMar-Pogo	33	7041	0.01%
DNA tran/TcMar-Fot1	4	2212	0.00%
DNA tran/hAT	23	14,996	0.03%
Total Class II repeat	280	556,784	1.37%
Unknown	2771	2,190,565	5.39%
Satellite	33	39,338	0.09%
Simple repeat	6665	307,146	0.75%
rRNA	92	204,174	0.50%
Low complexity	1152	64,801	0.15%
Rolling-circles	42	19,870	0.04%
Total repeat	12,544	5,834,277	14.35%

**Table 3:** Functional annotation of *P. ostreatus* genes from information in public databases.

Public Protein Database	Number of Genes	Percentage (%)
Nr	12,917	89.86%
Pfam	7980	55.52%
SwissProt	6459	44.94%
KOG	4217	29.34%
KEGG	4261	29.64%
Total	14,374	100.00%

**Table 4:** The gene distribution of different fungi in the six major modules of CAZymes.

Species	Total	GH	GT	AA	PL	CE	CBM
*Pleurotus ostreatus* PC80	634	246	52	169	53	31	83
*Pleurotus ostreatus* PC9	402	165	47	83	19	17	71
*Pleurotus ostreatus* PC15	554	240	64	118	29	22	81
*Pleurotus giganteus*	514	231	126	91	18	36	12
*Pleurotus tuoliensis*	322	150	51	51	24	29	17
*Pleurotus eryngii*	339	180	38	62	15	30	14
*Pleurotus placentodes*	434	184	38	97	17	10	88
*Pleurotus cystidiosus*	439	190	36	106	13	13	81

## Data Availability

The raw sequencing data and genome assembly have been stored in the National Center for Biotechnology Information (NCBI). The SRA accession number for the raw sequence data is SRR31742191. The accession number for the assembled genome is JBMSAL000000000.

## Supplementary Materials

The following supporting information can be downloaded at: https://www.mdpi.com/article/10.3390/jof11080563/s1, Table S1: GenBank accession numbers of pheromone receptors for phylogenetic analysis. Table S2: Scaffold size of *Pleurotus ostreatus* strain PC80. Table S3: Telomere sequence length and repeat count of *Pleurotus ostreatus* strain PC80. Table S4: Number of annotated genes in each scaffold of *Pleurotus ostreatus* strain PC80. Table S5: KEGG annotations and KOG/COG functional classifications in the genomes of *Pleurotus ostreatus* strains PC80, PC9_JGI, and PC15. Table S6: Numbers of carbohydrate-active enzymes in different *Pleurotus ostreatus* genomes. Table S7: The sequence size and structure of mating-type loci A and mating-type loci B in *Pleurotus ostreatus* strain PC80. Table S8: CDS sequence identity of *hd1* genes among PC9, PC15, and PC80. Table S9: CDS sequence identity of hd2 genes among PC9, PC15, and PC80. Table S10: Protein sequence identity of *hd1* genes among PC9, PC15, and PC80. Table S11: Protein sequence identity of *hd2* genes among PC9, PC15, and PC80. Table S12: Transcriptional expression (FPKM) of hd1 gene variants in *Pleurotus ostreatus* strain PC80. Table S13: Conserved domain prediction results of pheromone receptor genes in *Pleurotus ostreatus* strain PC80. Table S14: Transmembrane domains of the pheromone receptor in *Pleurotus ostreatus* strain PC80. Table S15: Amino acid sequence identity of pheromone receptor genes in PC80. Figure S1: GenomeScope model fitting and K-mer distribution characteristics with sequencing data for the *Pleurotus ostreatus* strain PC80. Figure S2: BUSCO analysis of genome completeness assessment in the *Pleurotus ostreatus* strain PC80. Figure S3: Telomere diagram of the genome in *Pleurotus ostreatus* strain PC80. The red dots at both ends of the scaffold represent telomere structures. Figure S4: BUSCO-based assessment of the annotation completeness of the genome in *Pleurotus ostreatus* strain PC80. Figure S5: NR Annotation of the Genome in *Pleurotus ostreatus* strain PC80. Figure S6: Genome collinearity between *Pleurotus ostreatus* strains PC80 and PC15. Figure S7: Structure of the mating-type locus A of in *Pleurotus ostreatus* strains PC9 and PC15. Figure S8: Comparison of *hd1* CDS sequences from PC9, PC15, and PC80. Figure S9: Comparison of *hd2* CDS sequences from PC9, PC15, and PC80. Figure S10: Protein sequence alignment of pheromone *php-1* and *php-2* in the *Pleurotus ostreatus* strain PC80. Figure S11: Protein sequence alignment of *PC80_php-s1* with *Pleurotus eryngii var. eryngii php3.2.* Figure S12: Protein sequence alignment of the pheromone receptor in the *Pleurotus ostreatus* strain PC80. Figure S13: Phylogenetic tree of pheromone receptors in the *Pleurotus ostreatus* strain PC80.
