# Supplementary material for: Chromosome-Level Genome Announcement of the Monokaryotic Pleurotus ostreatus Strain PC80
Source: J Fungi (Basel). 2025 Jul 29;11(8):563. doi: 10.3390/jof11080563 (PMC12387193; doi:10.3390/jof11080563)
Supplement: Supplementary file 1 [file jof-11-00563-s001.zip › Supplementary Figure.pdf]

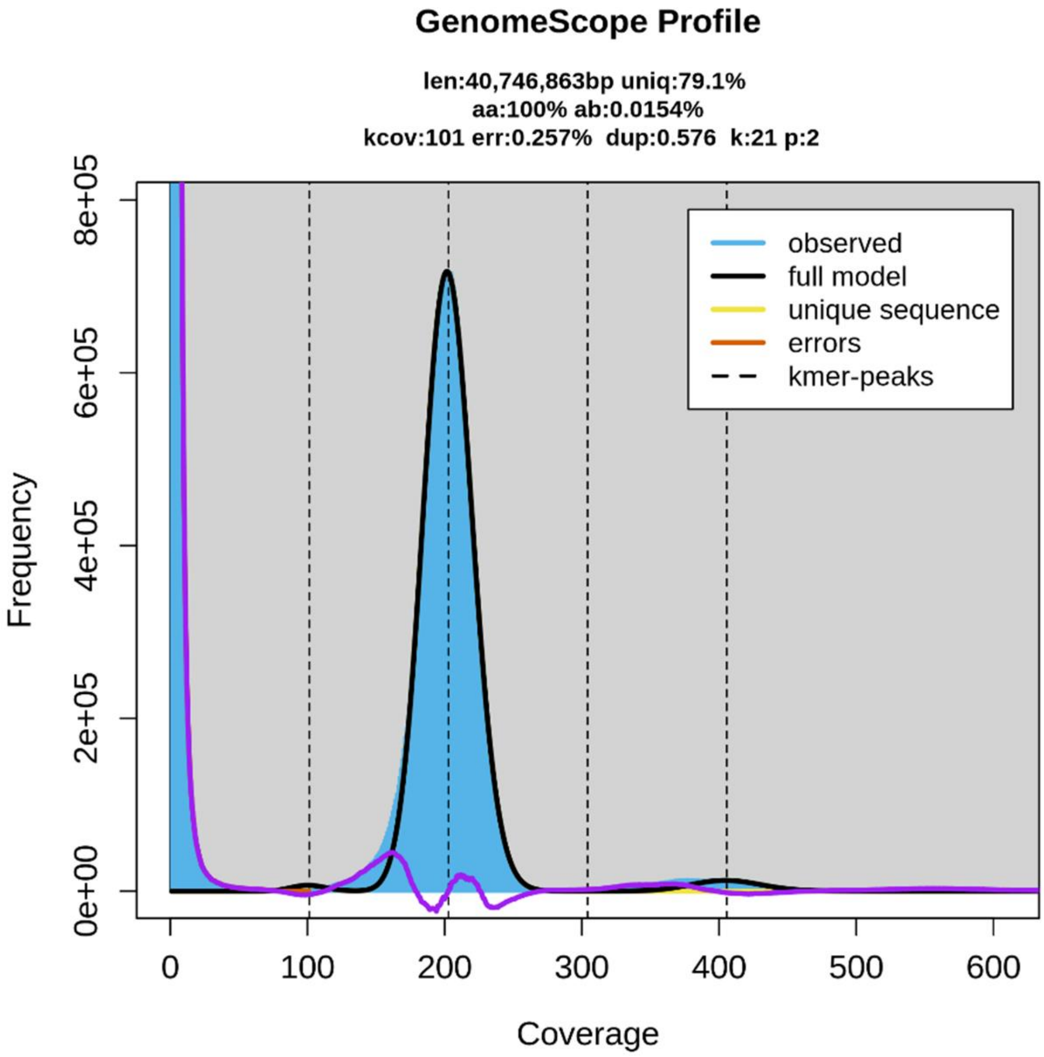

Supplementary Figure S1. GenomeScope model fitting and K-mer distribution characteristics with sequencing data for the *Pleurotus ostreatus* strain PC80.

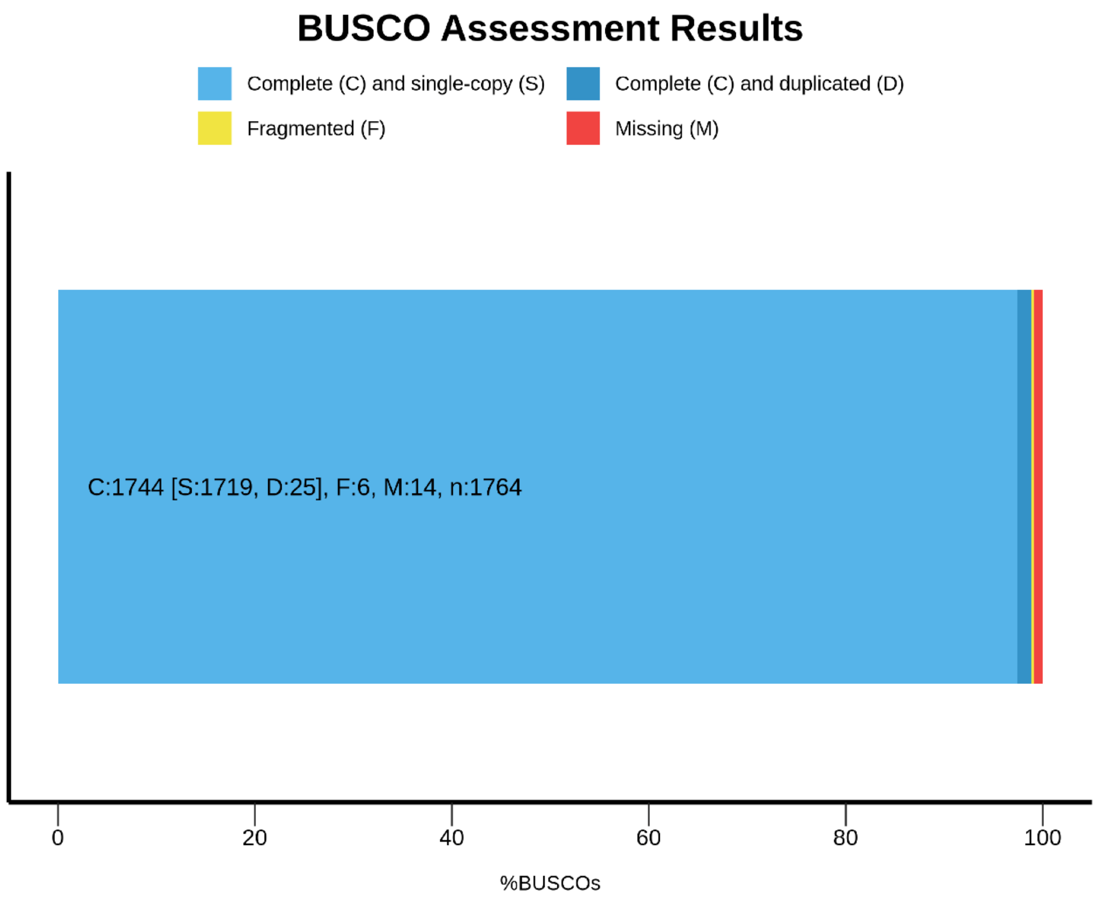

Supplementary Figure S2. BUSCO analysis of genome completeness assessment in the *Pleurotus ostreatus* strain PC80.

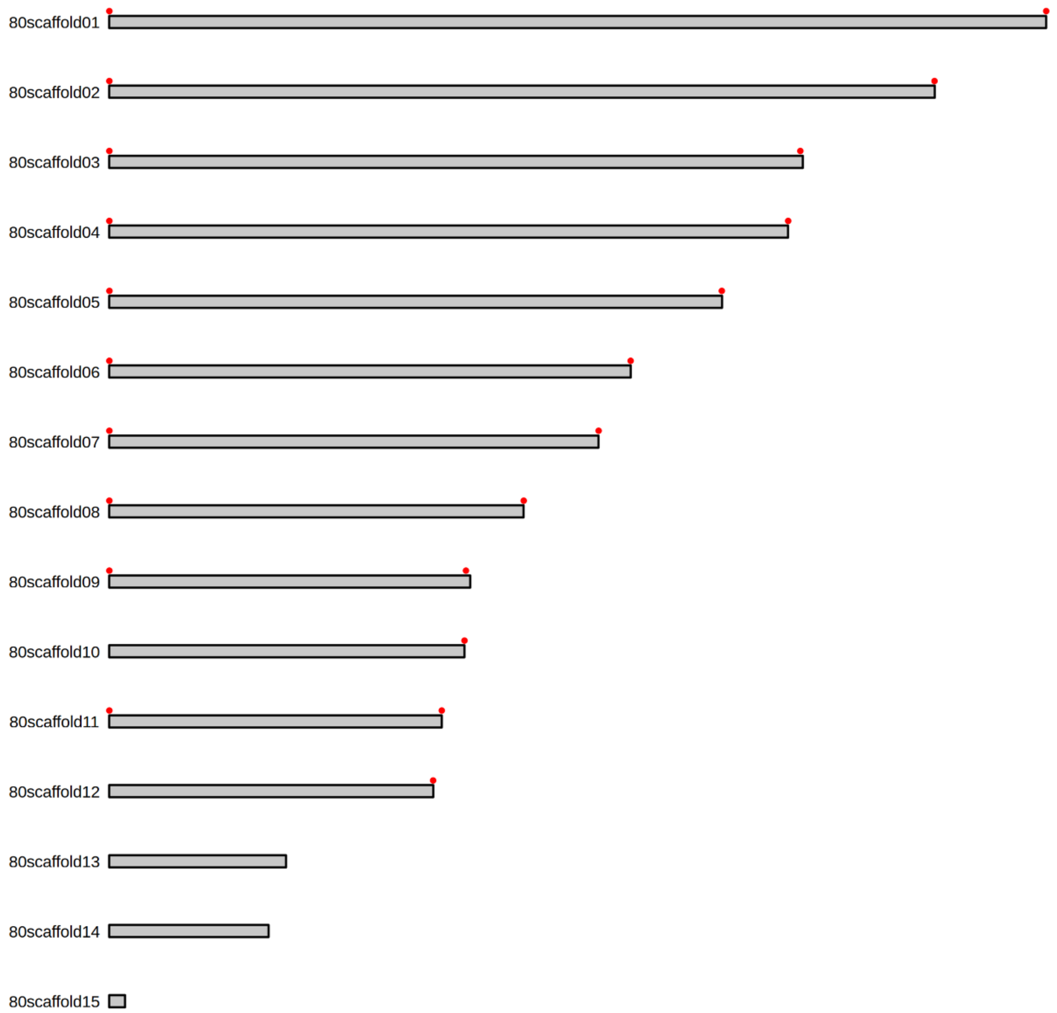

Supplementary Figure S3. Telomere diagram of the genome in *Pleurotus ostreatus* strain PC80. The red dots at both ends of the scaffold represent telomere structures.

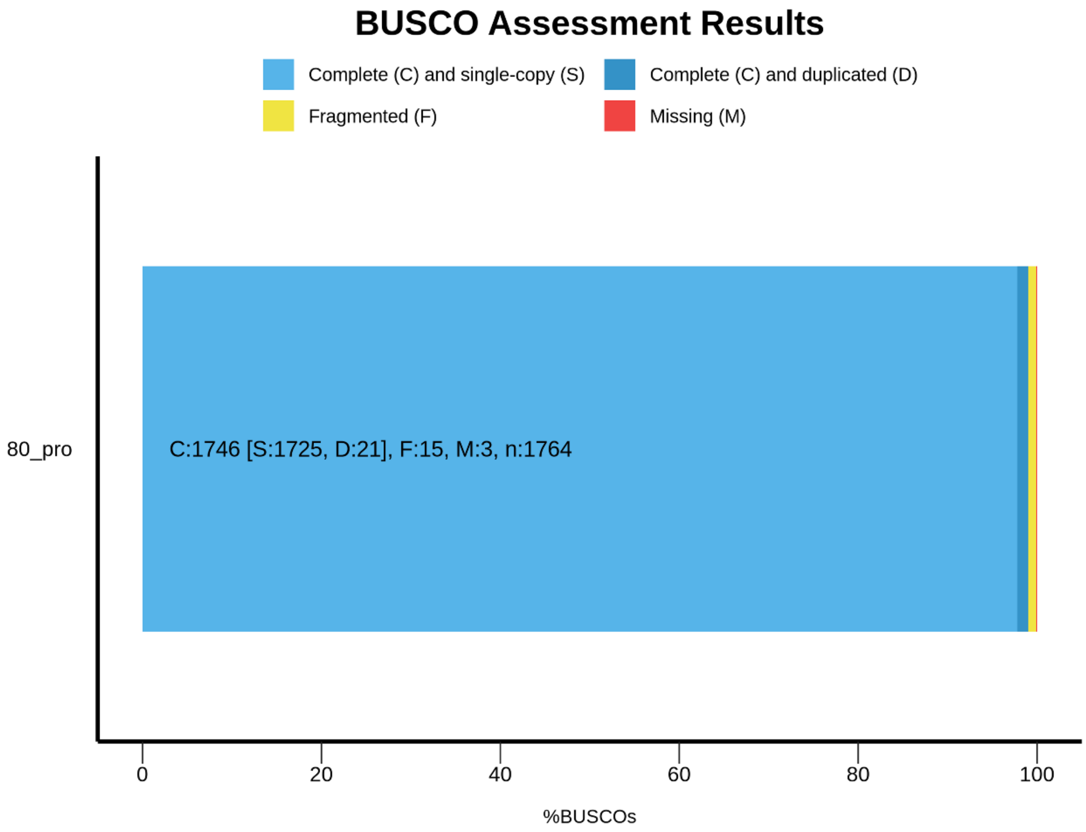

Supplementary Figure S4. BUSCO-based assessment of the annotation completeness of the genome in *Pleurotus ostreatus* strain PC80.

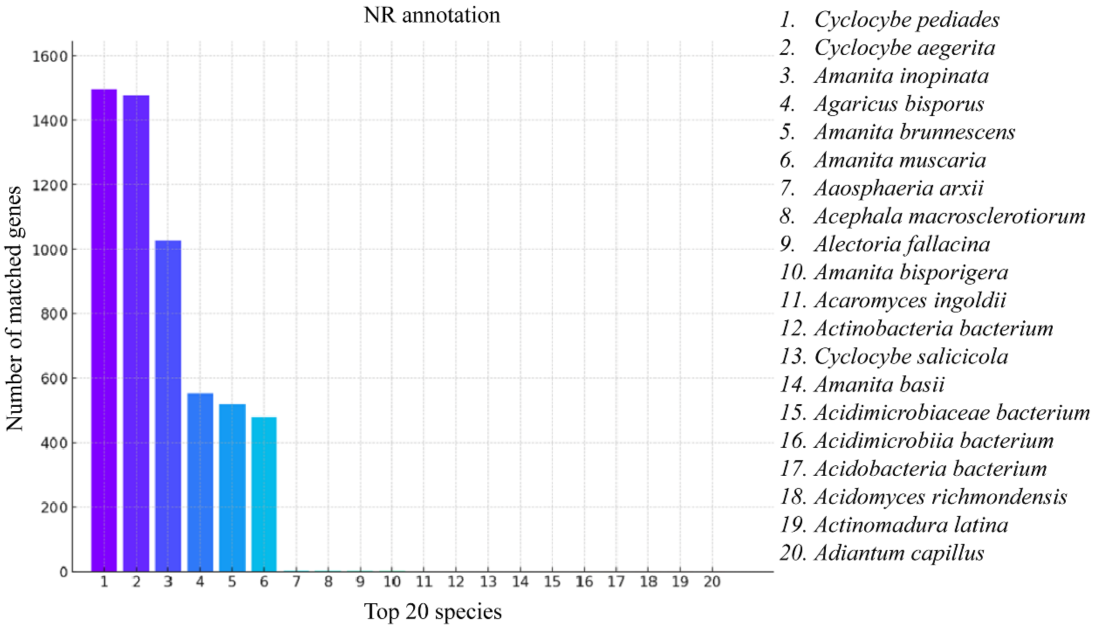

Supplementary Figure S5. NR Annotation of the Genome in *Pleurotus ostreatus* strain PC80.

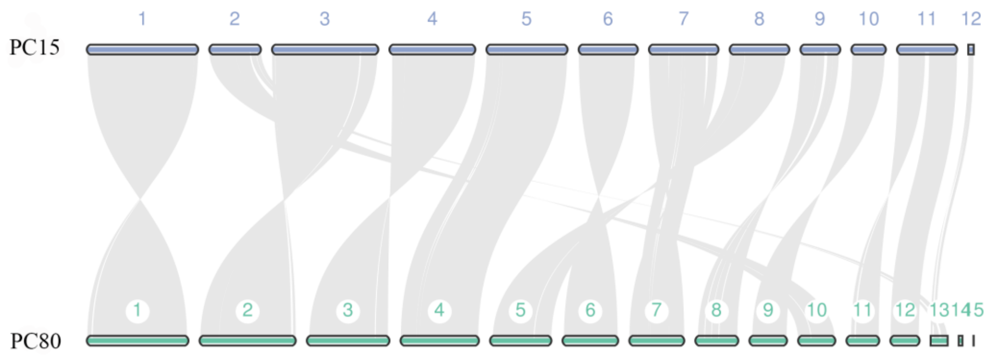

Supplementary Figure S6. Genome collinearity between *Pleurotus ostreatus* strains PC80 and PC15.

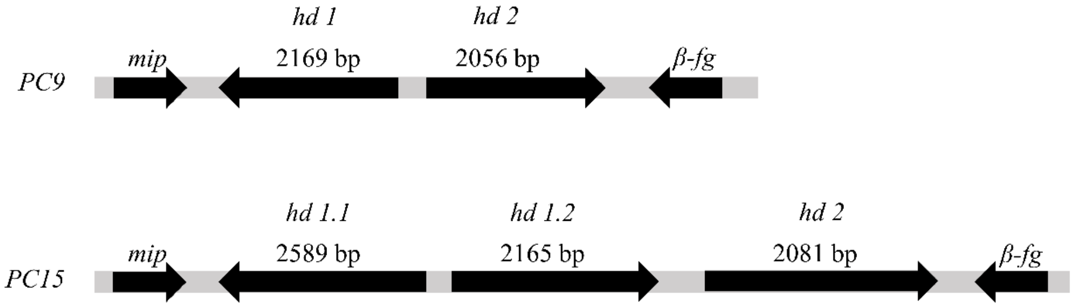

Supplementary Figure S7. Structure of the mating type locus A of in *Pleurotus ostreatus* strains PC9 and PC15.

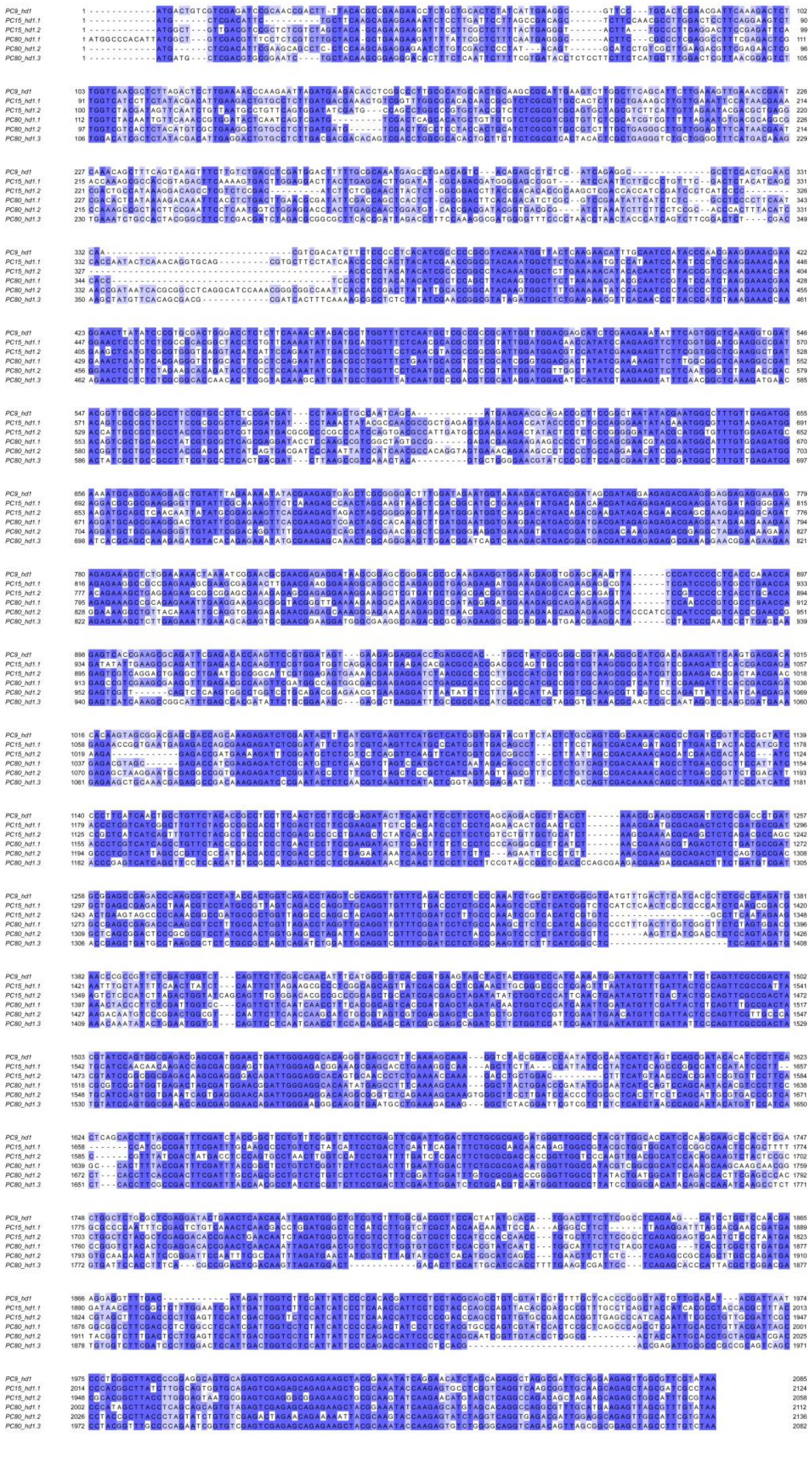

Supplementary Figure S8. Comparison of *hd1* CDS sequences from PC9, PC15, and PC80.

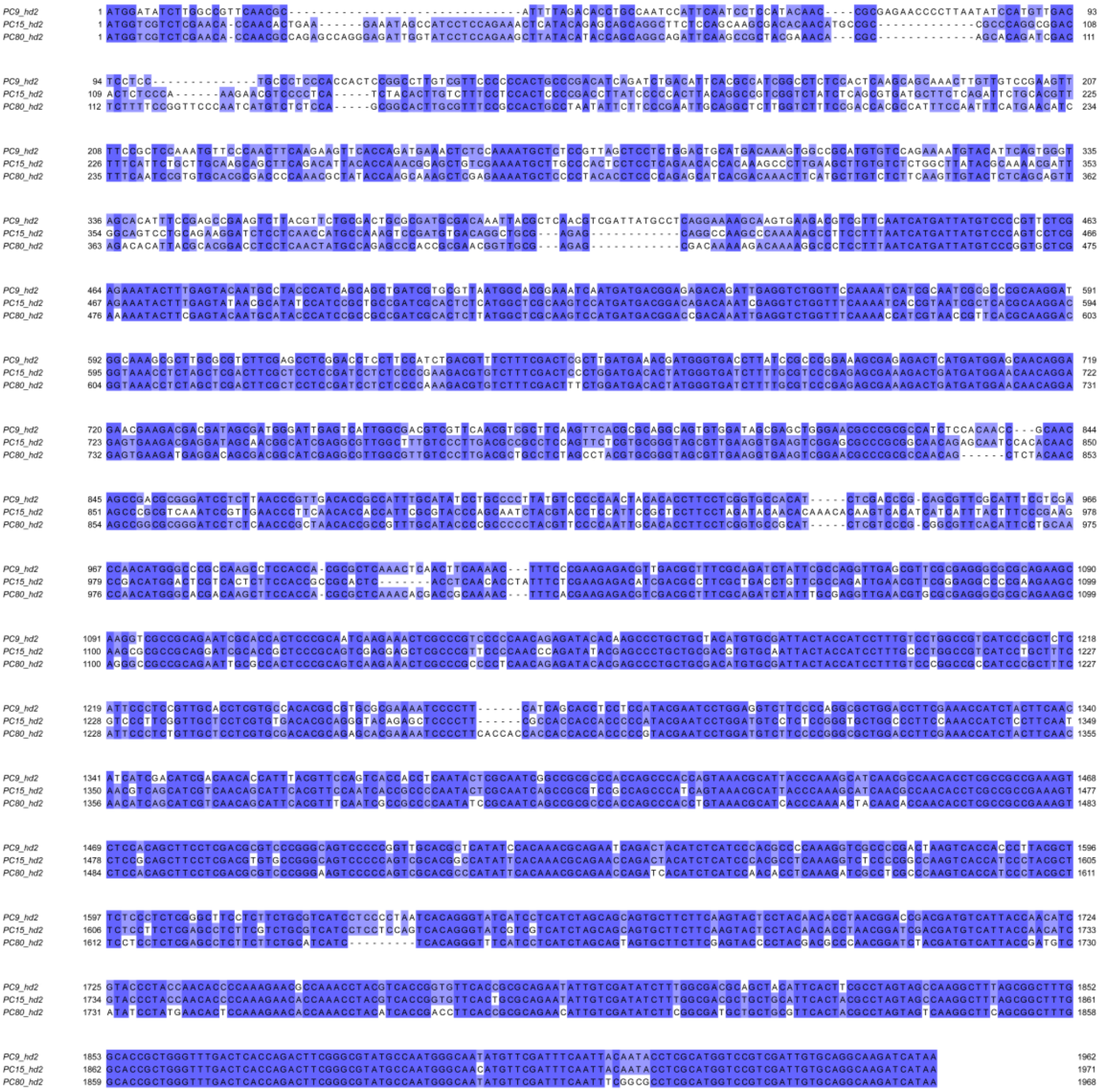

Supplementary Figure S9. Comparison of *hd2* CDS sequences from PC9, PC15, and PC80.

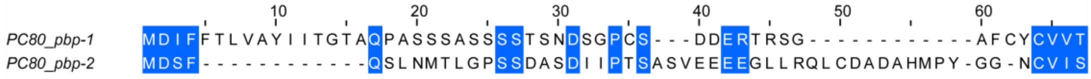

Supplementary Figure S10. Protein sequence alignment of pheromone *php-1* and *php-2* in the *Pleurotus ostreatus* strain PC80.

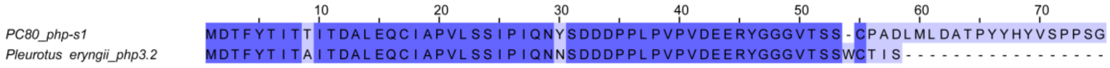

Supplementary Figure S11. Protein sequence alignment of *PC80_php-s1* with *Pleurotus eryngii* var. *eryngii php3.2*.

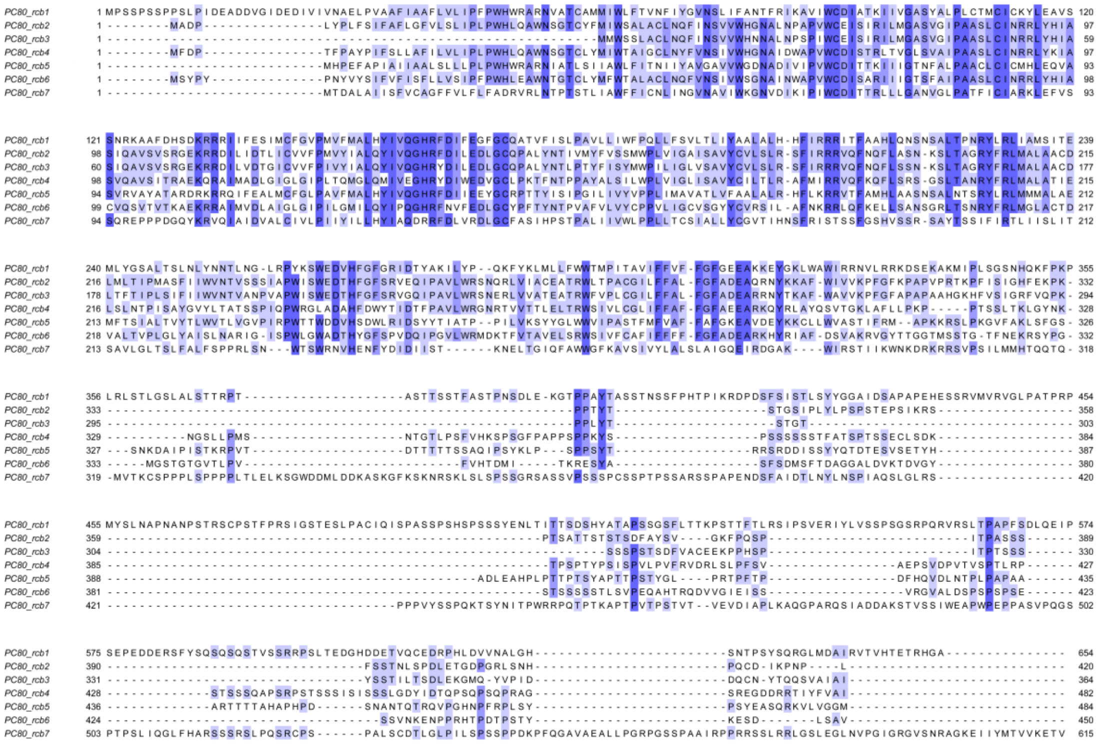

Supplementary Figure S12. Protein sequence alignment of pheromone receptor in the *Pleurotus ostreatus* strain PC80.

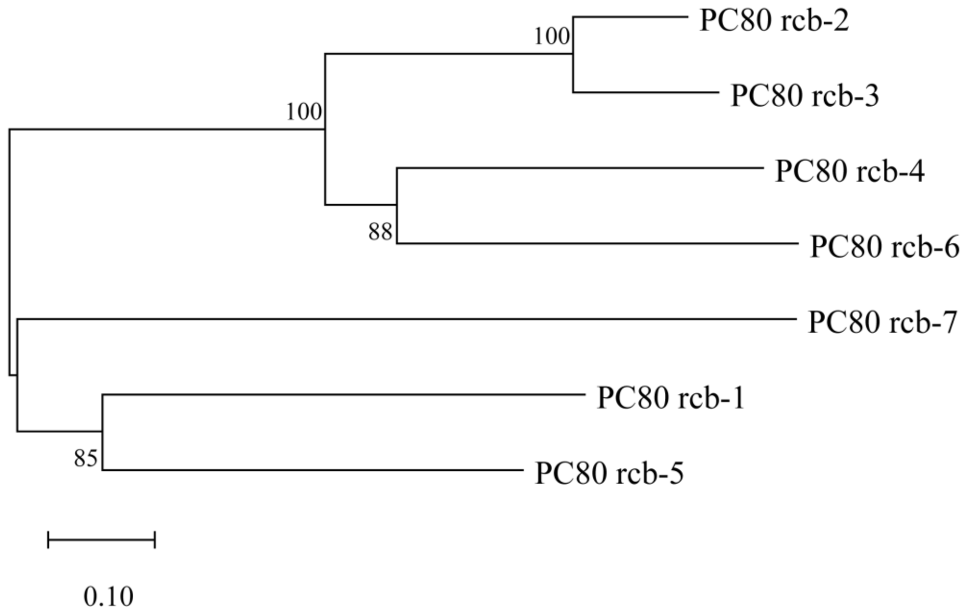

Supplementary Figure S13. Phylogenetic tree of pheromone receptors in the *Pleurotus ostreatus* strain PC80.
